# Competition of CO and Acetaldehyde Adsorption and Reduction on Copper Electrodes and Its Impact on *n*-Propanol Formation

**DOI:** 10.1021/acscatal.3c00190

**Published:** 2023-03-15

**Authors:** Alisson H. M. da Silva, Quentin Lenne, Rafaël E. Vos, Marc T. M. Koper

**Affiliations:** Leiden Institute of Chemistry, Leiden University, Leiden 2300 RA, The Netherlands

**Keywords:** propanol, copper, acetaldehyde, CO reduction, competition, ethylene

## Abstract

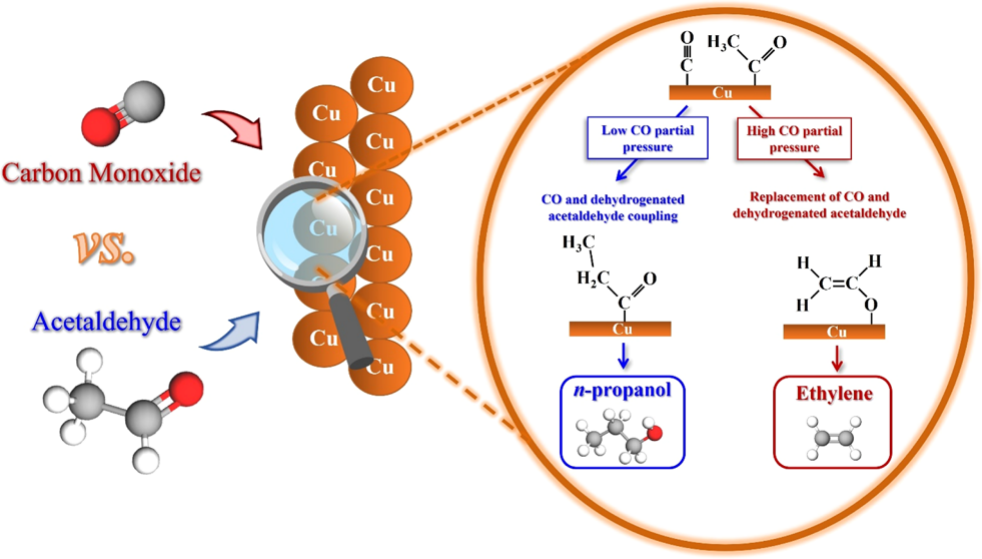

Selective synthesis of *n*-propanol from electrocatalytic CO_2_/CO reduction on copper remains challenging and the impact of the local interfacial effects on the production of *n*-propanol is not yet fully understood. Here, we investigate the competition between CO and acetaldehyde adsorption and reduction on copper electrodes and how it affects the *n*-propanol formation. We show that *n*-propanol formation can be effectively enhanced by modulating the CO partial pressure or acetaldehyde concentration in solution. Upon successive additions of acetaldehyde in CO-saturated phosphate buffer electrolytes, *n*-propanol formation was increased. Oppositely, *n*-propanol formation was the most active at lower CO flow rates in a 50 mM acetaldehyde phosphate buffer electrolyte. In a conventional carbon monoxide reduction reaction (CORR) test in KOH, we show that, in the absence of acetaldehyde in solution, an optimum ratio of *n*-propanol/ethylene formation is found at intermediate CO partial pressure. From these observations, we can assume that the highest *n*-propanol formation rate from CO_2_RR is reached when a suitable ratio of CO and acetaldehyde intermediates is adsorbed. An optimum ratio was also found for *n*-propanol/ethanol formation but with a clear decrease in the formation rate for ethanol at this optimum, while the *n*-propanol formation rate was the highest. As this trend was not observed for ethylene formation, this finding suggests that adsorbed methylcarbonyl (adsorbed dehydrogenated acetaldehyde) is an intermediate for the formation of ethanol and *n*-propanol but not for ethylene. Finally, this work may explain why it is challenging to reach high faradaic efficiencies for *n*-propanol, as CO and the intermediates for *n*-propanol synthesis (like adsorbed methylcarbonyl) compete for active sites on the surface, where CO adsorption is favored.

## 1 Introduction

The electrocatalytic CO_2_ reduction reaction (CO_2_RR) is considered as a promising approach to close the carbon cycle by converting CO_2_ into value-added chemicals and fuels driven by renewable electricity. Electrocatalysts have been identified with high efficiencies for the synthesis of the two-electron products CO^1−4^ and formate.^5−8^ However, lower conversion rates and selectivity are observed for more reduced products. Because the first studies from Hori,^9,10^ Cu is the most common metal used as the catalyst and it is still the only metal able to reduce CO_2_ into C_2+_ compounds with high efficiencies. While high current densities (1.6 A cm^–2^) for CO_2_ reduction to ethylene on copper have been recently reported,^11^ for C_3_ compounds (*n*-propanol, allyl alcohol, acetone, propionaldehyde, and hydroxyacetone), the selectivity and production rates remain rather low. Among them, *n*-propanol is the most favored C_3_ product, with Faradaic efficiencies (FEs) commonly between 3 and 15%.^12,13^

Different strategies have been applied to enhance the formation of the C_3_ compounds, especially *n*-propanol. The most common strategy is changing the electrode morphology. Following Hori’s works, different Cu surface morphologies have been investigated for CO_2_RR. High surface area Cu electrodes have shown an enhancement in the productivity of *n*-propanol.^12−16^ For example, at −0.95 V vs RHE, a Cu electrode covered with Cu nanoparticles with a roughness factor of 24 shows a current density for *n*-propanol of −24.7 mA cm^–2^ and about 11% of FE. On the other hand, on a conventional electropolished Cu disk, with a roughness factor defined as 1, *n*-propanol formation corresponds to a current density of only −2.1 mA cm^–2^ and about 1.5% of FE.^13^ Using highly fragmented copper structures as the electrode, Pang et al.^17^ showed that *n*-propanol could be formed from CO reduction with 20% of FE at −8.5 mA cm^–2^. Enhancement of the activity toward *n*-propanol is also commonly achieved using alloying strategies. A Pd–Cu foam (Pd_9_Cu_91_) electrode was shown to form *n*-propanol with 13.7% of FE, corresponding to a current density of −1.15 mA cm^–2^ at −0.65 V vs RHE.^12^ More recently, Wang et al. showed that performing CO reduction instead of CO_2_ reduction on an Ag–Ru–Cu electrode results in 36% of FE to *n*-propanol and −300 mA cm^–2^ of total current density.^18^

The strategy to use CO instead CO_2_, as applied by Wang et al.,^18^ has led to the idea to apply a two-step cascade process in which the first step consists of converting CO_2_ into CO followed by its conversion into *n*-propanol in a second step. Wu et al.^19^ showed that a tandem system consisting of two electrolyzers for converting CO_2_ to CO and CO to *n*-propanol, resulted in a FE of 15.9% for *n*-propanol. Similarly, Romero Cuellar et al.^20^ showed that the total FE toward multi-carbon products of a one-step system using the Cu gas diffusion electrode as a working electrode was limited to 20% at a total current density of −470 mA cm^–2^ while the two-step configuration [using a Ag-gas diffusion electrode (GDE) as an electrode for the first step followed by Cu-GDE in the second step] led to a total FE toward C_2_ and C_3_ products of 62% (with about 18% of *n*-propanol) at a total current density of −300 mA cm^–2^.

Although this strategy is an interesting approach to enhance the *n*-propanol formation, understanding the local effects impacting the productivity of *n*-propanol is also important for optimizing future experimental design. For example, it is not well understood how the intermediates interact with each other and how their local concentrations influence the *n*-propanol formation. It has been reported that *n*-propanol is formed from the coupling between adsorbed CO and an adsorbed methylcarbonyl (dehydrogenated acetaldehyde) intermediate (H_3_C–CO*).^22,23^ It has also been suggested that *n*-propanol is formed via CO* trimerization (CO–CO–CO),^24^ which is then further reduced to the alcohol. Even if both pathways would occur simultaneously, they would depend differently on local concentrations. In this context, the acetaldehyde pathway would be the more likely pathway if an increase in the local concentration of acetaldehyde enhances *n*-propanol formation. On the other hand, the CO trimerization pathway to *n*-propanol would be the favored pathway to *n*-propanol if its formation increases with increasing CO* coverage but would be hindered by the presence of acetaldehyde.

In this work, we evaluate how the local concentrations of CO and acetaldehyde impact the *n*-propanol formation by changing either the concentration of acetaldehyde added to the electrolyte or the CO flow rate (and CO partial pressure) during the electrolysis. We find that the local concentrations of both acetaldehyde and CO impact the *n*-propanol formation. In summary, *n*-propanol formation is affected positively when a higher concentration of acetaldehyde is present in the electrolyte with a constant CO flow rate, or with a constant acetaldehyde concentration when a lower CO flow rate is applied. In the absence of acetaldehyde in solution, an optimum ratio of *n*-propanol/ethylene is found at intermediate CO partial pressure. From these observations, we can assume that the optimal *n*-propanol formation rate is reached at a suitable ratio of CO and adsorbed acetaldehyde intermediates. The CO* trimerization pathway seems rather unlikely on the basis of the results presented here. Moreover, under the same conditions, a maximum *n*-propanol formation rate is reached when ethanol formation is minimal, suggesting that ethanol and *n*-propanol share methylcarbonyl as an intermediate.

## 2 Methods

### 2.1 Cleaning Procedure

Ultrapure water (resistivity >18.2 MΩ cm, TOC <5 ppb) was used for all experiments in this work. Before each day of measurements, the glassware and the homemade PEEK H-cell used in this work were soaked in a beaker completely submerged in an acid solution of permanganate (0.5 M H_2_SO_4_ + 1 g L^–1^ KMnO_4_) for at least 12 h. Then, the H-cell and the glassware were drained and rinsed with a dilute piranha solution [1:3 v/v of H_2_O_2_ (Merck, Emprove exp)/H_2_SO_4_] to remove residual KMnO_4_ and MnO_*x*_. Afterward, the H-cell and the glassware were again drained, rinsed with ultrapure water, and finally boiled (in ultrapure water) for at least three times.

### 2.2 Electrode Preparation

A Cu disk electrode (1 cm^2^, 99.99%, Matek) was first mechanically polished on a microcloth (Buehler) with diamond suspensions (Buehler) of 3.0, 1.0, and 0.25 μm successively. Next, the electrode was rinsed and sonicated in ultrapure water for at least 10 min to remove remnant diamond suspension on the surface. Then, the electrode was electropolished in H_3_PO_4_ (85%) holding the potential at 3 V vs graphite (as a counter electrode) for 30 s. The electrode was rinsed with ultrapure water to remove remnants of the H_3_PO_4_ solution on the surface. Finally, Cu was electrodeposited on the electropolished Cu disk, applying −10 V vs Cu (as a counter electrode) for 60 s in 0.1 M H_2_SO_4_ solution containing 0.1 M CuSO_4_ resulting in a 100 mA cm^–2^ current density. Scanning electron microscopy images of the resulting electrode surface are shown in Figure S1.

### 2.3 Electrochemistry

All electrochemical experiments were carried out in the homemade PEEK H-cell (total 15 mL) in a three-electrode configuration. A dimensionally stable anode (DSA, Magneto Special Anodes) was used as a counter electrode. The DSA was separated from the working electrode (Cu, as prepared before) using a Nafion 117 membrane (Aldrich), for neutral pH, and an AHMV membrane (AGC) for alkaline pH. Mini HydroFlex (Gaskatel) was used as a reference electrode. All reported potentials were recorded vs. the reversible hydrogen electrode (RHE) scale. All potentials were controlled with an Ivium potentiostat (Ivium Technologies). Resistances were determined via impedance spectroscopy (EIS) and 85% ohmic drop compensation was applied during the experiment.

Voltammograms were recorded from −0.1 to −1.2 V vs RHE, with a 50 mV s^–1^ scan rate, in a 0.1 M potassium phosphate buffer solution (pH = 7), prepared using potassium dihydrogen phosphate (KH_2_PO_4_ 99.99%, Merck) and dibasic potassium phosphate (K_2_HPO_4_ 99.99%, Merck). Phosphate buffer was chosen as an electrolyte rather than bicarbonate buffer to prevent aldol condensation as bicarbonate is an alkaline solution in the absence of CO_2_ (pH = 8.2) and it does not act as a buffer when CO_2_ is not continuously purged in the solution. Regarding experiments with acetaldehyde, different concentrations of acetaldehyde (>99.5%, Sigma-Aldrich) were added to the buffer electrolyte. For the tests with CO, CO (>99.99%, Lindegas) was bubbled into the buffer electrolyte for at least 10 min to guarantee the complete saturation of the solution before the start of the measurement.

CO reduction was carried out in the H-cell filling up each compartment with 7.5 mL of electrolyte. Before each measurement, CO was bubbled through the electrolyte for at least 15 min to reach CO saturation. CORR, with and without initial acetaldehyde in a potassium phosphate buffer electrolyte, was carried out at fixed potentials until the conversion charge reached 30 coulombs. Tests of drag vaporization of *n*-propanol were performed by purging argon in a 10 mM *n*-propanol solution for an hour and no strong evaporation was observed when different flow rates were applied. CORR in 0.1 M KOH was carried out at fixed potentials for 1 h. During the electrolysis, a constant CO flow was provided using a mass flow controller (Brooks). For the partial pressure experiments, argon (5.0, Lindegas) was used as the second gas and two mass flow controllers were used to provide different flow compositions. The gas products from the electrolysis were analyzed using gas chromatography (Micro-GC, Agilent), equipped with two thermal conductivity detectors (TCD), both using He as a carrier gas. A CP-SIL 5B column was used to separate CO_2_, CH_4_, and C_2_H_4_ on one TCD, while the combination of MS5A and CP-PORABOND Q columns were used to separate H_2_, O_2_, N_2_, CH_4_, and CO on the other TCD. Liquid products were analyzed via using high-performance liquid chromatography (HPLC, Shimadzu) with an Aminex HPX-87H column (BioRad) equipped with an RID detector.

## 3 Results and Discussion

**Figure 1:**
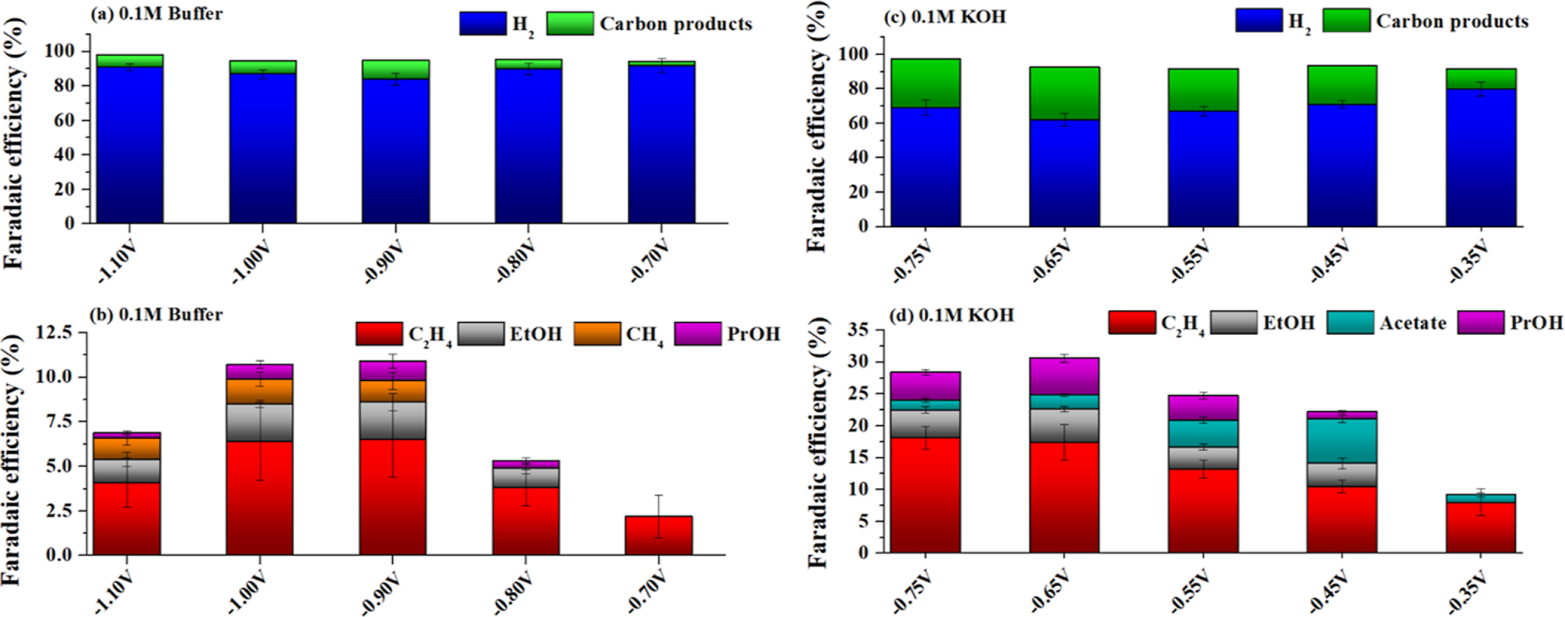
FEs to H_2_ (blue bars), total carbon products (green bars), C_2_H_4_ (red bars), ethanol (blue bars), *n*-propanol (green bars), acetate (pink bars), and CH_4_ (orange bars) in (a,b) 0.1 M potassium phosphate buffer (pH = 7) and (c,d) 0.1 M KOH (pH = 13), both saturated with CO.

Figure 1 shows the FE for hydrogen, methane, ethylene, acetate, ethanol, and *n*-propanol from CO reduction in a 0.1 M potassium phosphate buffer electrolyte (pH = 7) and in 0.1 M KOH (pH = 13). While FE toward C_2+_ compounds can reach over 30% in 0.1 M KOH, the maximum FE to C_2+_ compounds was limited to 10% at −0.9 V vs RHE in the phosphate buffer electrolyte, with C_2_H_4_ as the main carbon product. For *n*-propanol, the maximum FE was found to be around 1% at −0.9 V in the buffer electrolyte, while 6% was found at −0.65 V in KOH. It is known that the formation of C_2+_ compounds is enhanced in alkaline pH due to the favored formation of the CO dimer.^25−27^ Thus, it is reasonable that higher FE for C_2+_ compounds are observed in KOH than in potassium phosphate buffer. Moreover, phosphate anions have been reported to act as proton donors thereby enhancing the hydrogen evolution reaction (HER).^28^ Indeed, higher FEs for H_2_ are observed in Figure 1a in the potential range evaluated in this work. On the other hand, the buffer electrolyte appears as a good choice for tests with aldehydes. The reaction pathway for *n*-propanol formation has been reported to involve the coupling between CO and methylcarbonyl.^21−23^ Because aldehydes undergo aldol condensation at alkaline pHs,^29,30^ controlled testing with aldehydes is only reliable using a neutral buffer solution. Furthermore, the production of *n*-propanol is low when only CO reduction occurs in the buffer electrolyte. Then, an improvement in *n*-propanol formation due to the addition of acetaldehyde and its coupling with CO can be easily detected. Thus, for the experiments involving acetaldehyde in solution, a potassium phosphate buffer (pH = 7) was used as an electrolyte, unless otherwise indicated.

**Figure 2:**
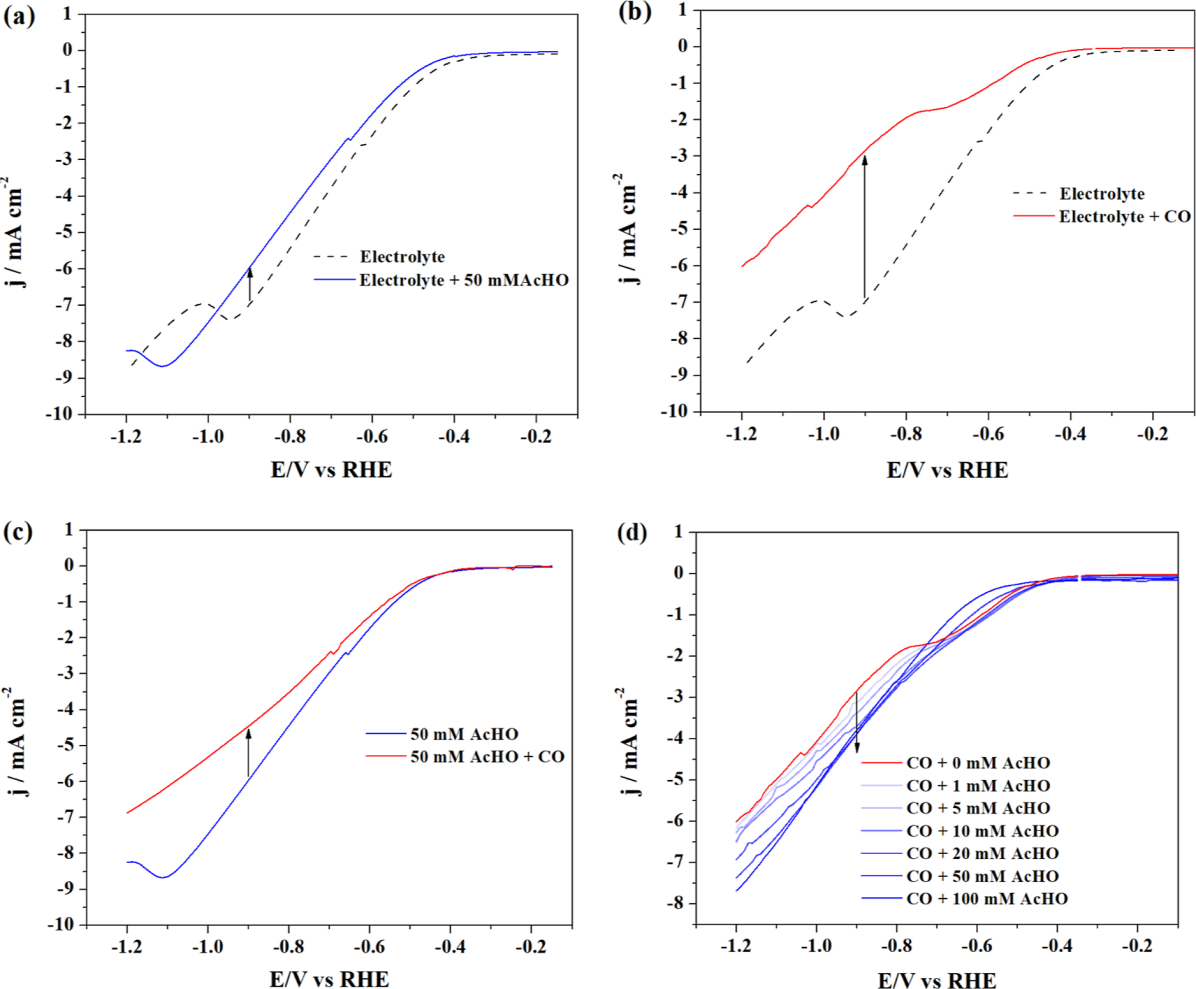
LSV between −0.1 and −1.2 V vs RHE of (a) potassium phosphate buffer (black dashed line) and after 50 mM of acetaldehyde was added (blue line); (b) potassium phosphate buffer (black dashed line) and after CO was saturated in the electrolyte (red line); (c) 50 mM of acetaldehyde in the buffer electrolyte (blue line) and after CO was saturated in the acetaldehyde solution (red line); and (d) CO-saturated buffer electrolyte (red line) and after acetaldehyde were added to the electrolyte in different concentrations (blue lines). Scan rate: 50 mV/s. Each curve represents the third LSV of that measurement and, therefore, 6 LSVs were recorded for graphs (a–c) and 21 were recorded for graph (d).

The competition between CO and acetaldehyde adsorption and reduction was first evaluated by linear sweep voltammetry (LSV). Figure 2 shows the voltammograms (Figure 2a–d) when CO (Figure 2a) or acetaldehyde (Figure 2b) is added to the buffer electrolyte. Figure 2c depicts the differences observed upon CO saturation of a buffer solution containing acetaldehyde, while the opposite procedure, the progressive addition of acetaldehyde to a CO-saturated buffer solution, is shown in Figure 2d. Figure 2a,b shows similar trends: in absolute numbers, the current density decreases when CO or acetaldehyde is added to the buffer electrolyte. As in the absence of CO or acetaldehyde only the HER takes place, Figure 2a,b indicates that HER is partially suppressed when CO or acetaldehyde is present. CO or acetaldehyde compete with the water and biphosphate reduction for the same active sites, reducing the activity for HER. The suppression of HER is observed to be stronger in the presence of CO as the decrease in the current density is more pronounced than the decrease observed upon acetaldehyde addition. For example, when 50 mM acetaldehyde was added to the electrolyte, we observe a current density loss of 1 mA cm^–2^ at −0.9 V (from −6.95 to −5.9 mA cm^–2^), while a decrease of ∼4 mA cm^–2^ is observed when CO is saturated in the electrolyte (from −6.95 to −2.85 mA cm^–2^). By comparison, CO-saturated water has a CO concentration of ∼1 mM at standard temperature and pressure (CO solubility = 27.6 mg L^–1^ or 0.986 mM).^31^ In other words, although CO has a 50 times lower concentration, the current density decreases over 2 times more than in the presence of acetaldehyde. Figure 2c,d shows the competition between CO and acetaldehyde. Figure 2c shows that the current density drops significantly when CO is added to the electrolyte containing 50 mM of acetaldehyde (from −5.9 to −4.4 mA cm^–2^). The opposite test was also done by adding different amounts of acetaldehyde in the CO-saturated electrolyte (Figure 2d). At −0.9 V, a current increase of 0.2 mA cm^–2^ is observed when 1 mM of acetaldehyde is added. When 100 mM of acetaldehyde is added to the CO-saturated solution, an increase of 1 mA cm^–2^ is observed—from −2.85 to −3.9 mA cm^–2^. As both CO and acetaldehyde are competing for the same active sites on the Cu surface, the results shown in Figure 2c,d strongly indicate that CO is preferably adsorbed and reduced on the surface compared to acetaldehyde.

We expect that the competition between CO and acetaldehyde adsorption can affect the *n*-propanol formation from CO_2_RR. As mentioned before, various literature reports have confirmed that acetaldehyde and its adsorbed counterpart (methylcarbonyl) is a key intermediate for the formation of *n*-propanol.^21−23^ If methylcarbonyl formed during CO_2_RR is desorbed due to the competition with other adsorbates, such as formed CO, it will be unavailable to react with CO to produce *n*-propanol. Figure S2 shows the acetaldehyde conversion and ethanol production rate after 1 h of electrolysis at −0.9 V when 50 mM acetaldehyde is reduced in the absence and in the presence of CO. Acetaldehyde conversion is 23% in the absence of CO and 14% in the presence of CO. The ethanol production rate in the presence of CO is measured considering both CORR and acetaldehyde reduction, as both form ethanol as a product. Even considering the contribution of ethanol formation from CORR, the total ethanol production is smaller: it decreased from 94 μmol cm^–2^ h^–1^ in the absence of CO to 62 μmol cm^–2^ h^–1^ in the presence of CO. Thus, as discussed before, acetaldehyde reduction is directly affected by the presence of (adsorbed) CO.

**Figure 3:**
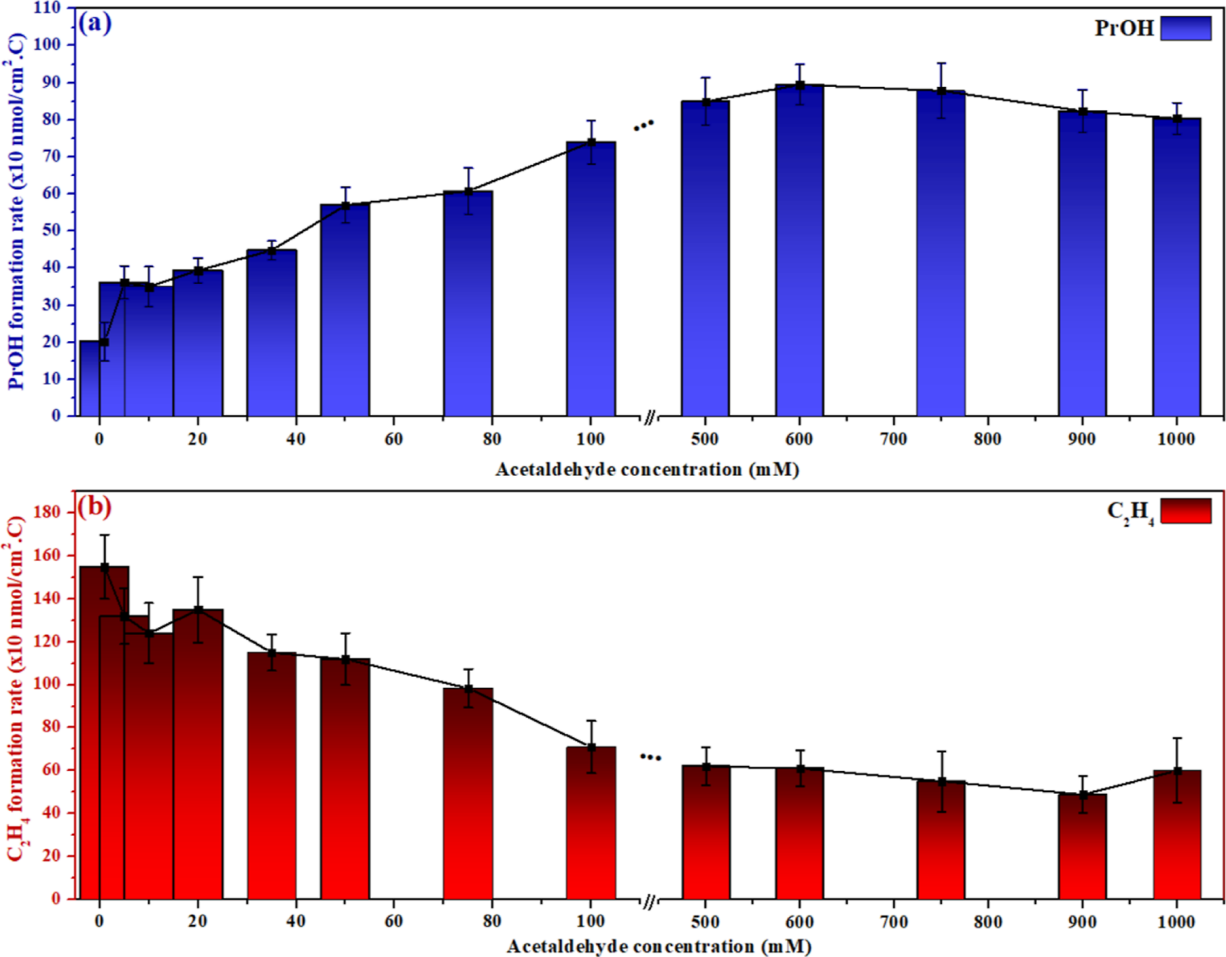
(a) *n*-Propanol formation rate and (b) ethylene formation rate when different concentrations of acetaldehyde were added to the CO-saturated buffer electrolyte before the electrolysis started.

**Figure 4:**
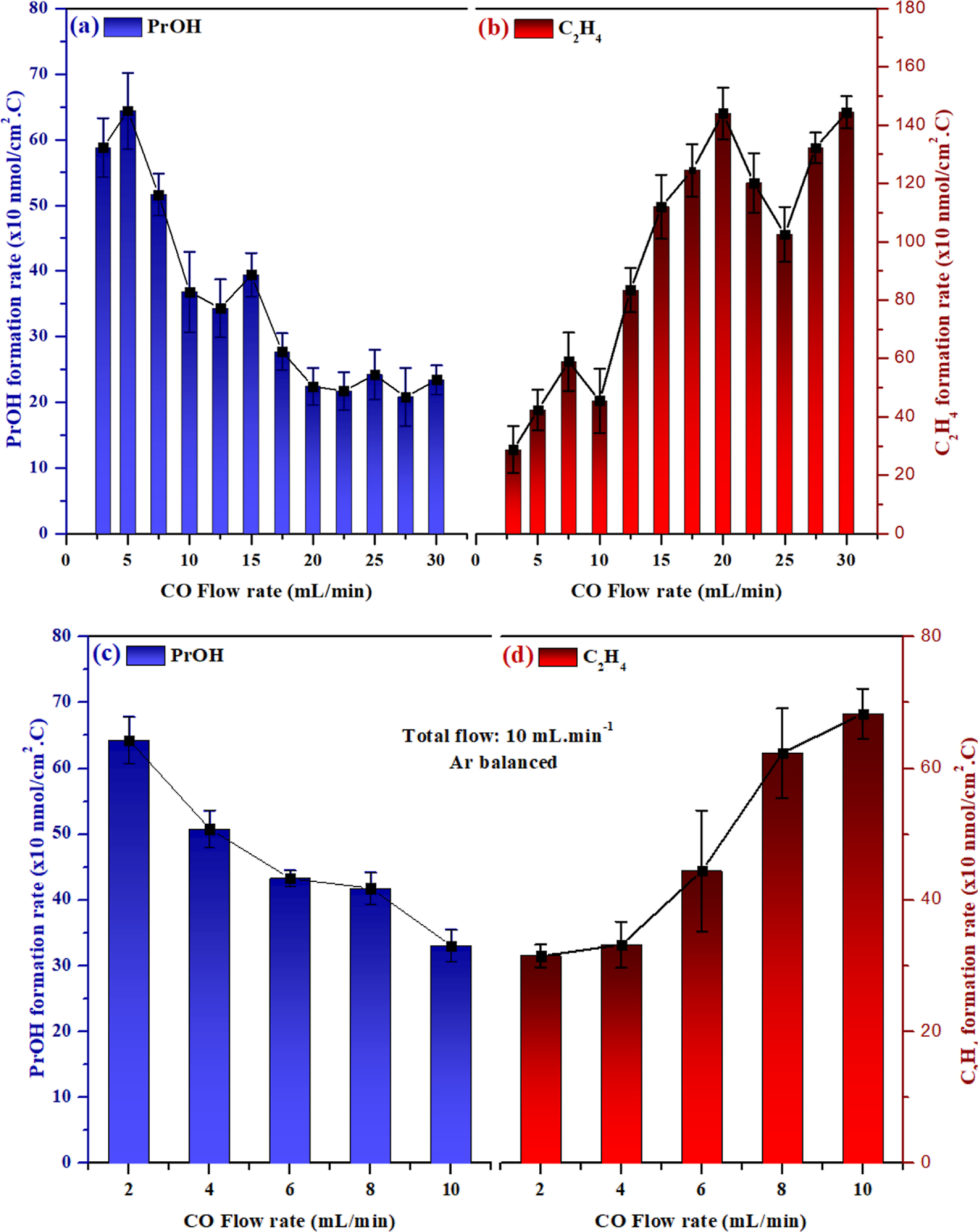
Formation rate of (a,c) *n*-propanol and (b,d) ethylene for different CO flow rates with (a,b) different total flow or with (c,d) partial pressure experiments (with same total flow—10 mL min^–1^, Ar balanced). Electrolyte: 50 mM acetaldehyde in 0.1 M potassium phosphate buffer.

To enhance the *n*-propanol formation, an equal mixture of acetaldehyde and CO should adsorb to reach the ideal molar composition (CO* + CH_3_CO* + 6H^+^ + 6e^–^ → CH_3_CH_2_CH_2_OH + H_2_O). To evaluate how *n*-propanol formation is affected by the local concentration of CO and acetaldehyde, two approaches were used as follows: (i) increasing the local concentration of acetaldehyde or (ii) decreasing the local concentration of CO. These approaches are illustrated in Figures 3 and 4. Figure 3a shows how the *n*-propanol formation rate changes when different concentrations of acetaldehyde are added to the CO-saturated buffer electrolyte. For this experiment, CO was purged continuously (15 mL min^–1^) through the cell during the electrolysis to keep the electrolyte saturated. The *n*-propanol formation rate (Figure 3a) increases with the concentration of acetaldehyde until a plateau is observed for acetaldehyde concentrations above 100 mM. This saturation is also observed in Figure 2d, where the current density is not strongly affected anymore when acetaldehyde concentration is increased from 50 to 100 mM. As both acetaldehyde and CO compete for active sites on the surface, if more acetaldehyde adsorbs and/or react, less CO can react to form ethylene and a decreasing in ethylene production should be observed, as is confirmed in Figure 3b. The decline in ethylene production could be interpreted by the amount of acetaldehyde concentration added to the electrolyte that dominates the reaction to form, mainly, ethanol and therefore less CO would be able to adsorb to form ethylene. However, a decline in the CO/acetaldehyde coupling does not happen; in fact, the opposite is observed. Therefore, the interpretation that ethylene formation only decreases because the surface sites are increasingly engaged in acetaldehyde reduction to (mainly)ethanol would not fit for *n*-propanol formation. If we consider that *n*-propanol is formed via different pathways than via methylcarbonyl–CO coupling, the *n*-propanol formation rate should also decline similarly to that observed for ethylene. As the opposite was observed (Figure 3), the only explanation we found reasonable to our results was considering that *n*-propanol is formed via methylcarbonyl–CO coupling and this step was enhanced because a better balance of methylcarbonyl and CO was reached on the electrode surface (supported by LSVs in Figure 2)

Figure 4 shows how the *n*-propanol and ethylene formation change with changing CO flow rates at a constant initial acetaldehyde concentration of 50 mM. Figure 4a shows that with a higher CO flow rate, a lower *n*-propanol production rate is observed. A plateau is observed for flow rates over 20 mL min^–1^, indicating that the local concentration of CO reached its maximum. It is important to mention that the flow rate where the plateau is observed may vary when electrodes of different surface areas are used as this will affect the interfacial CO consumption. At lower flow rates, the local concentration of CO might be lower as the CO transport from the bulk to the surface can be limiting. In agreement with Figures 3b, Figure 4b also shows that the production rate for ethylene is higher when the local CO concentration is higher. However, mass transport of other species such as phosphate might also influence these results. To verify the trends from Figure 4a,b, partial pressure tests were performed, as shown in Figure 4c,d. Here, CO was mixed with Ar to keep the total flow constant at 10 mL min^–1^. Similar trends are observed for *n*-propanol and ethylene formation, confirming that CO depletion close to the surface is an important phenomenon for *n*-propanol formation. Figures 3 and 4 show that by either increasing the acetaldehyde concentration or by decreasing the CO flow rate, a better balance of acetaldehyde and CO molecules near the surface can be achieved, thereby promoting the formation of *n*-propanol.

**Figure 5:**
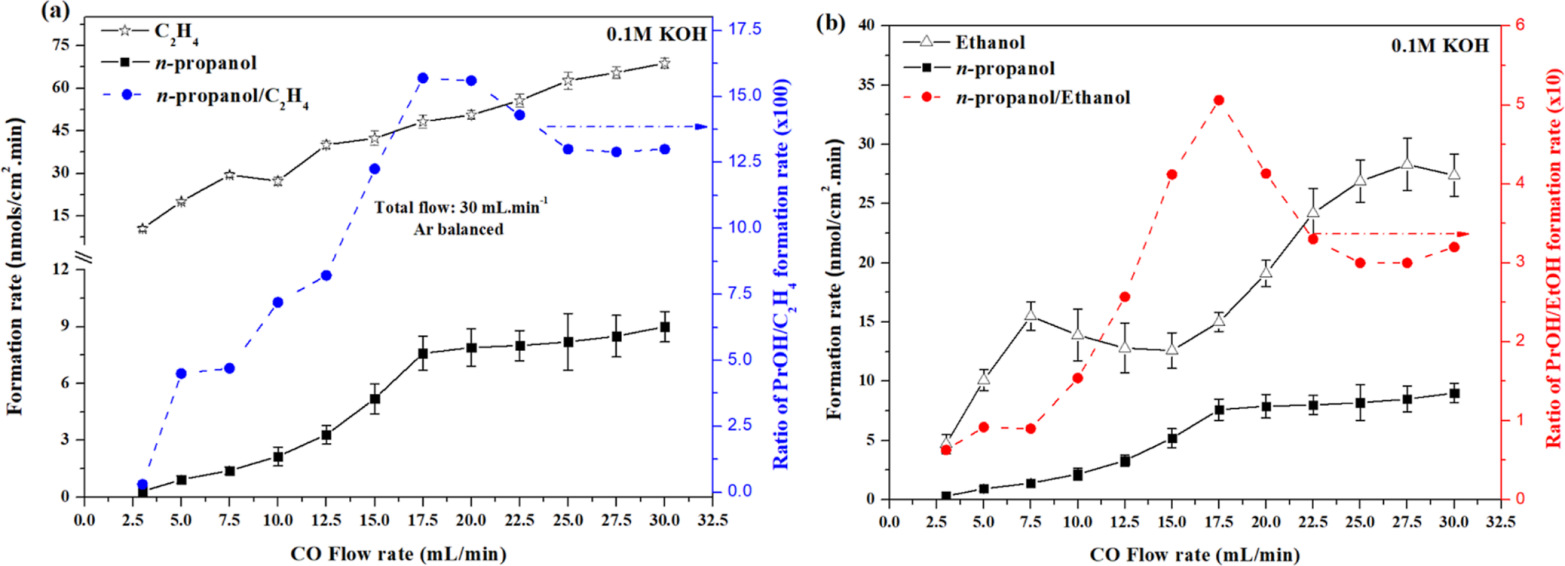
Formation rate vs CO flow rate in 0.1 M KOH for (a) ethylene (black line with stars markers); (b) ethanol (black line with triangles markers); and (a,b) *n*-propanol (black lines with squares markers).

The results shown above may explain why it is challenging to reach high FE for *n*-propanol. With low CO partial pressure, HER is favored and the FE to carbon products is compromised (Figure S3). At high CO partial pressure, the competition between CO and acetaldehyde favors CO adsorption and thus acetaldehyde conversion is compromised (Figure 4). To show this phenomenon under more realistic conditions, Figure 5 shows how the CO partial pressure affects the *n*-propanol, ethylene, and ethanol formation in CORR in 0.1 M KOH as an electrolyte (in the absence of acetaldehyde). The increase in CO partial pressure leads to an enhancement of *n*-propanol, ethylene, and ethanol formation rates, where the highest CO partial pressure results in the most efficient formation of all compounds. However, although the increase in CO partial pressure leads to an increase in the formation of the products, the ratios of *n*-propanol/ethylene (Figure 5a dashed blue line) and *n*-propanol/ethanol (Figure 5b dashed red line) formation rates show an optimum (under the current experimental conditions at 17.5 mL min^–1^ of CO, Ar balanced to 30 mL min^–1^ of total flow). Again, it is important to mention that the optimum flow rate may change depending on the electrode morphology, as it will affect the local consumption rate. This trend would be difficult to explain by a CO* trimerization pathway but would be expected if methylcarbonyl is formed during CORR and serves as an intermediate for *n*-propanol synthesis. The ethanol formation rate shows a minimum between 12.5 and 17.5 mL min^–1^ of CO flow while the propanol formation rate increases significantly, reaching its maximum at 17.5 mL min^–1^. The fact that the production of ethanol is minimal when the production of *n*-propanol reaches its maximum suggests that methylcarbonyl is an intermediate for the formation of both molecules. This trend is not observed for ethylene formation (Figure 5a), indicating that methylcarbonyl is not an intermediate in the ethylene pathway. Finally, our results show that low flow rates are not beneficial as a high local CO concentration is needed to form the key CO–dimer and the corresponding C_2_ intermediates, such as methylcarbonyl.^21−23^ Recently, Hou et al.^32^ have showed that the increase in CO coverage, by increasing the CO pressure, favors the selectivity toward oxygenates, especially acetate, over ethylene. In addition to the work of Hou et al., our results in Figure 5 also show that a too high CO flow rate is not advantageous as CO will induce the desorption of methylcarbonyl, leading to a lower likelihood of CO–methylcarbonyl coupling to form *n*-propanol, at the expense of a (relatively) higher ethylene production.

**Figure 6:**
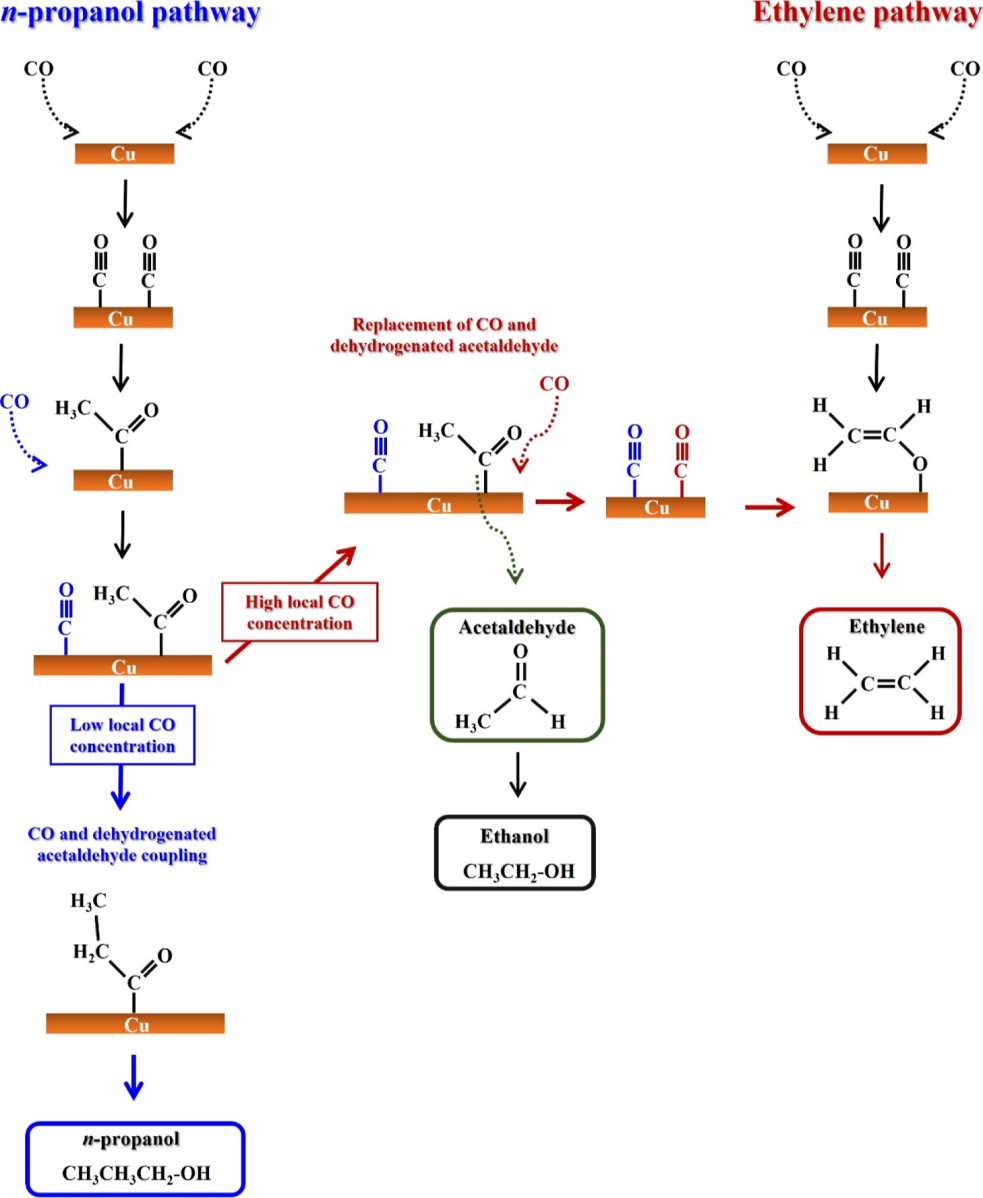
Reaction pathway to ethanol, acetaldehyde, ethylene, and *n*-propanol from CORR. Ethylene is favored at high CO local concentrations (red arrows) and *n*-propanol is favored at lower CO local concentrations (blue arrows). The way the adsorbates are represented here is based on previous works.^23,27,33,34^

A scheme summarizing the pathway to form *n*-propanol and ethylene is shown in Figure 6. The scheme does not represent all the steps because that is not the aim of this work. A more detailed reaction mechanism can be found elsewhere.^23,27,33,34^ The way how the adsorbates are represented here is based on previous works.^23,27,33,34^ However, it is worth mentioning that different ways on how the species adsorb on the Cu surface might also happen.

## 4 Conclusions

Competition between acetaldehyde and CO adsorption/reduction impacts the *n*-propanol formation during CO_2_ and CO reduction on copper electrodes. By increasing the acetaldehyde concentration in a CO-saturated phosphate buffer electrolyte, greater *n*-propanol formation is observed. On the other hand, the highest *n*-propanol formation rate was observed at the lowest CO flow rate applied in a 50 mM acetaldehyde phosphate buffer electrolyte. Through voltammetry and electrolysis experiments, we showed that CO inhibits acetaldehyde adsorption and conversion. When the local concentration of CO is decreased or that of acetaldehyde is increased, a better balance of acetaldehyde and CO molecules near the surface can be achieved and, thus, the formation of *n*-propanol is better promoted. In a conventional 0.1 M KOH electrolyte (without acetaldehyde in solution), the production of ethanol was minimal at intermediate CO partial pressure (17.5 mL min^–1^ of CO + 12.5 mL min^–1^ of Ar) while *n*-propanol production reached its maximum, indicating that methylcarbonyl (dehydrogenated acetaldehyde) is an intermediate for the formation of both molecules. This tendency was not observed for ethylene formation, suggesting that ethylene and *n*-propanol do not share methylcarbonyl as an intermediate. Moreover, as an optimum for *n*-propanol/ethylene formation was found at intermediate CO partial pressure, the CO* trimerization pathway to *n*-propanol synthesis seems rather unlikely at the conditions used in this work. Finally, the results shown here may explain why it is challenging to reach high FEs for *n*-propanol from CO_2_RR/CORR: at low CO flow rates, HER is favored to the detriment of carbon products; while at high CO flow rates, the competition between CO and acetaldehyde’s adsorption favors CO adsorption and thus the coupling between acetaldehyde and CO is compromised.

## Supporting Information Available

SEM images of the Cu electrode surface, acetaldehyde conversion in the absence and in the presence of CO, and Faradaic efficiency for H_2_ and carbon products at different CO partial pressures (PDF)
