# Supplementary material for: Competition of CO and Acetaldehyde Adsorption and Reduction on Copper Electrodes and Its Impact on n-Propanol Formation
Source: ACS Catal. 2023 Mar 15;13(7):4339–47. doi: 10.1021/acscatal.3c00190 (PMC10088027; doi:10.1021/acscatal.3c00190)
Supplement: Supplementary file 1 — cs3c00190_si_001.pdf [file cs3c00190_si_001.pdf]

# Supporting Information for:

# Competition of CO and acetaldehyde adsorption and reduction on copper electrodes and its impact on *n*-propanol formation


Alisson H. M. da Silva, Quentin Lenne, Rafaël E. Vos, Marc T. M. Koper*

Leiden Institute of Chemistry, Leiden University, Leiden, Netherlands.

**AUTHOR INFORMATION**

**Corresponding author**

Marc T.M. Koper – Leiden Institute of Chemistry, Leiden University, 2300 RA Leiden, The Netherlands. ORCID ID: https://orcid.org/0000-0001-6777-4594 ,

*E-mail:* m.koper@lic.leidenuniv.nl

**Authors:**

Alisson H. M. da Silva – Leiden Institute of Chemistry, Leiden University, 2300 RA Leiden, The Netherlands; ORCID ID: https://orcid.org/0000-0003-3198-3110

Quentin Lenne – Leiden Institute of Chemistry, Leiden University, 2300 RA Leiden, The Netherlands; ORCID ID: https://orcid.org/0000-0002-1849-6302

Rafaël E. Vos – Leiden Institute of Chemistry, Leiden University, 2300 RA Leiden, The Netherlands; ORCID ID: https://orcid.org/0000-0003-1810-1179

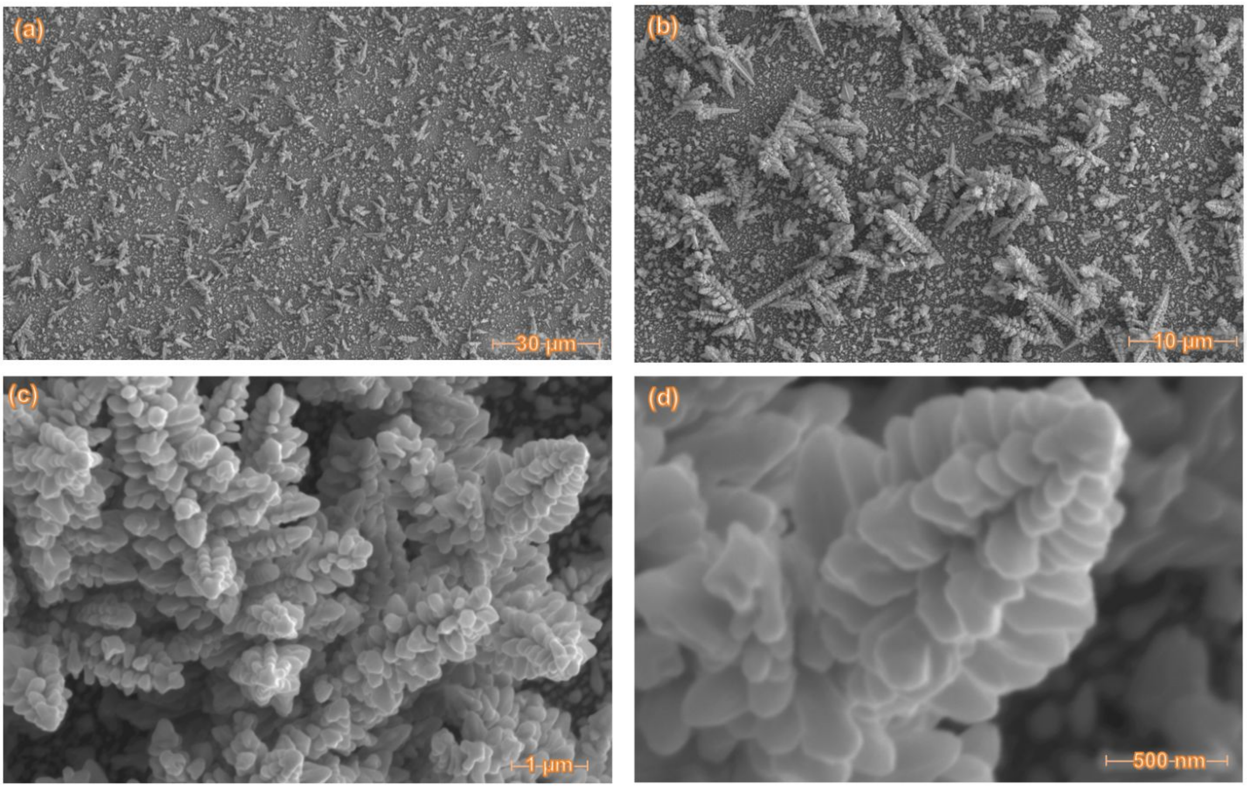


**Figure S1.** SEM images of Cu electrode surface after Cu electrodeposition. (a), (b), (c), and (d) are images of the nanostructures at different magnifications.

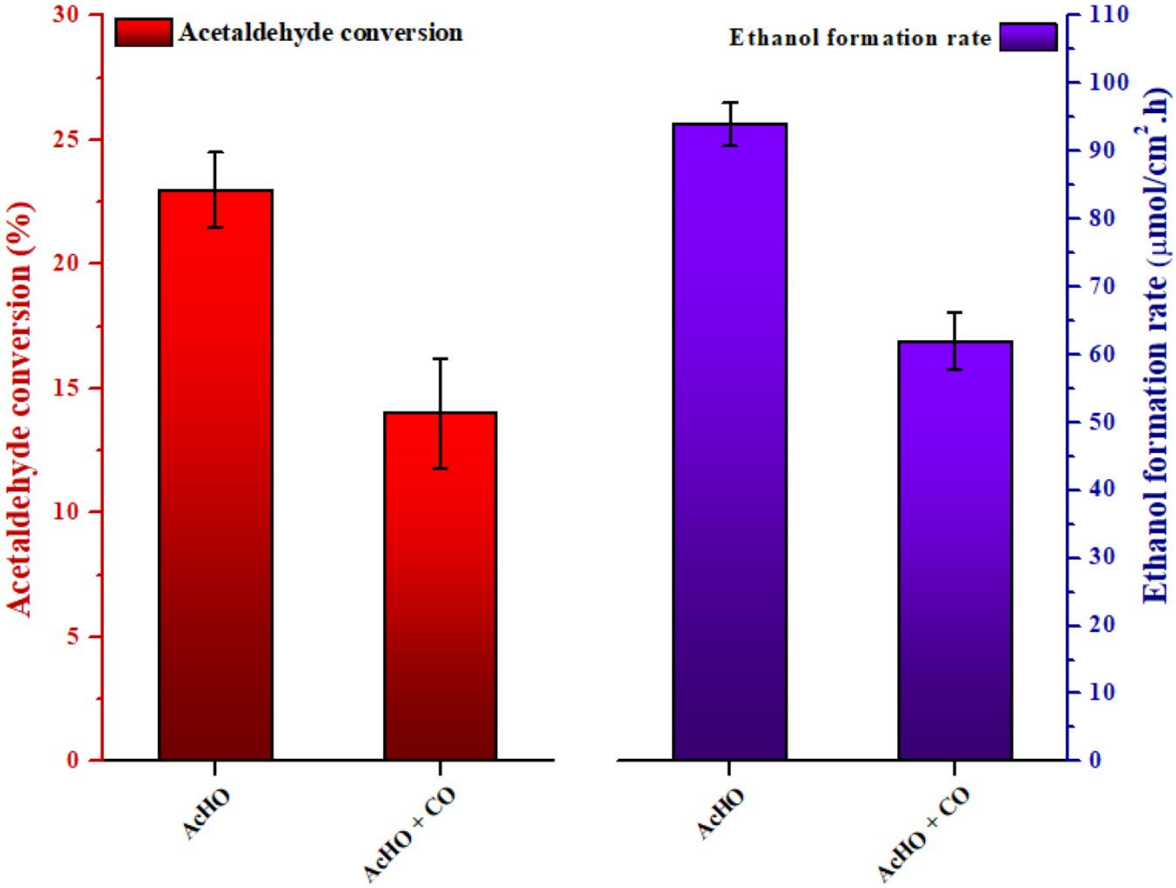


**Figure S2.** Acetaldehyde conversion in the presence and absence of CO (red bars) and ethanol formation rate in the presence and absence of CO (purple bars). Electrolyte: 50 mM acetaldehyde in 0.1M potassium phosphate buffer.

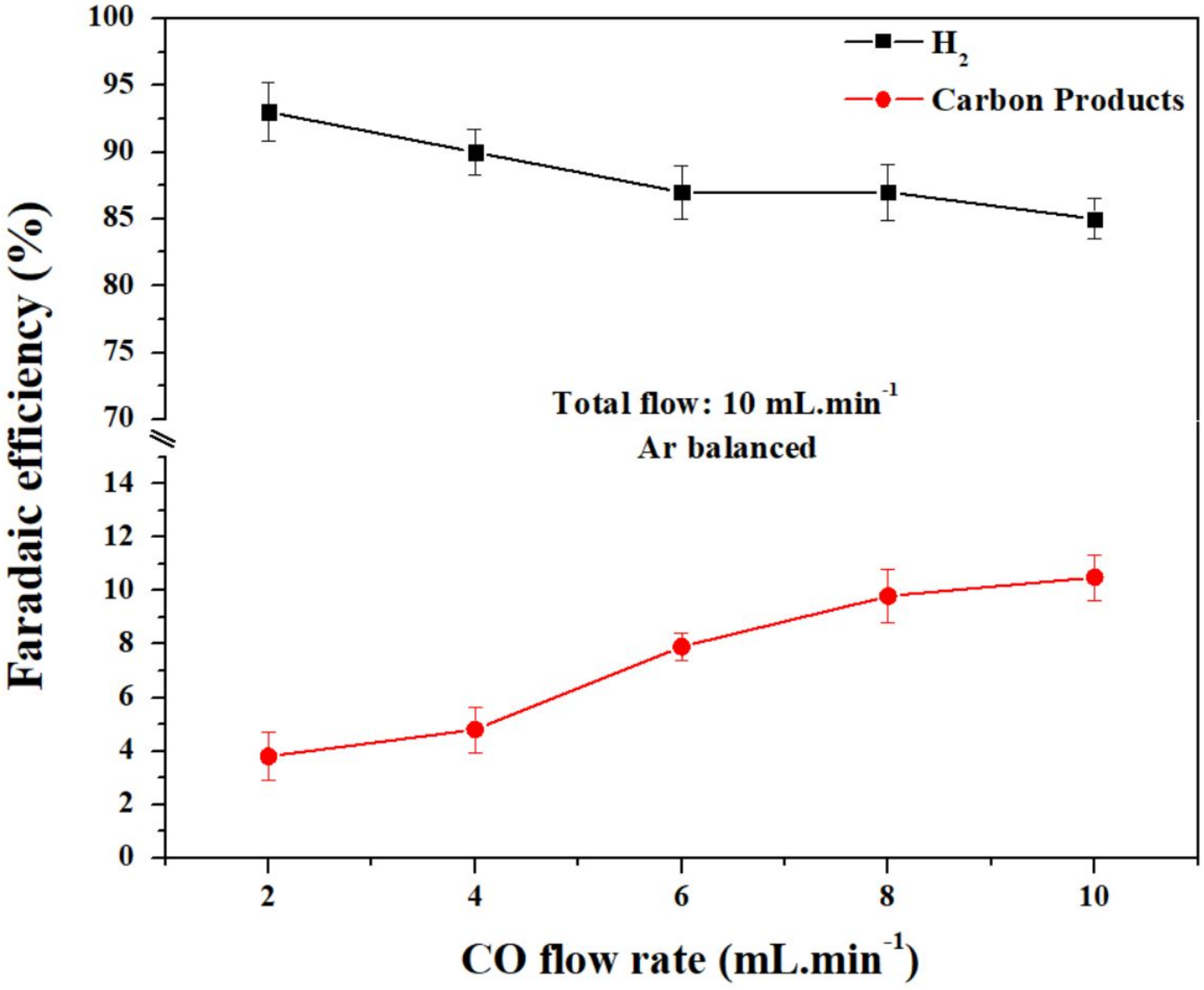


**Figure S3.** Faradaic efficiency for $H_2$ (black line) and carbon products (red line) in 0.1M potassium phosphate buffer at different CO partial pressure balanced with Argon.
